# Supplementary material for: MetaRibo-Seq measures translation in microbiomes
Source: Nat Commun. 2020 Jun 29;11:3268. doi: 10.1038/s41467-020-17081-z (PMC7324362; doi:10.1038/s41467-020-17081-z)
Supplement: Supplementary file 10 — Supplementary Data 7 [file 41467_2020_17081_MOESM10_ESM.zip › File2/Confidence_High_Taxonomy/143465_out.krona.html]

Javascript must be enabled to view this page.

members
magnitude
magnitudeUnassigned
count
unassigned
taxon
rank

143465\_out

4

superkingdom
2
4

1239
3
phylum

186801
3
class

order
186802
3

family
31979
1

genus

SRS971276\_contig\_number\_25224
580596
1

2
216572
family

459786
2
genus

species
2
1945593

SRS053356\_contig\_number\_3627SRS104400\_contig\_number\_57402

phylum
201174
1

1760
1
class

1
85010
order

2070
1
family

genus
1884922
1

1

SRS1041032\_contig\_number\_8565
1872711
species
